# Between scents and sterols: Cyclization of labdane-related diterpenes as model systems for enzymatic control of carbocation cascades

**DOI:** 10.1016/j.jbc.2024.108142

**Published:** 2024-12-26

**Authors:** Reuben J. Peters

**Affiliations:** Roy J. Carver Department of Biochemistry, Biophysics & Molecular Biology, Iowa State University, Ames, Iowa, USA

**Keywords:** terpene synthase, terpene cyclases, terpenoids, enzymatic mechanism, enzyme evolution

## Abstract

The citrus scent arises from the volatile monoterpene limonene, whose cyclic nature can be viewed as a miniaturized form of the polycyclic sterol triterpenoids. In particular, these rings are all formed from poly-isoprenyl precursors *via* carbocation cascades. However, the relevant reactions are initiated by distinct mechanisms, either lysis/ionization of an allylic diphosphate ester bond, as in limonene synthases, or protonation of a terminal olefin or epoxide, as in lanosterol synthases. Labdane-related diterpenoids are unique in their utilization of both types of reactions. With over 7000 such natural products known, this pair of reactions clearly generates privileged scaffolds, hydrocarbon backbones from which biological activity is readily derived. Moreover, the relevant enzymes serve as model systems for terpene cyclization more generally. Indeed, investigation of their enzymatic structure-function relationships has highlighted the importance of catalytic base positioning within the active site cavity in specifying product outcomes. Conversely, comparison to the cyclases for other types of terpenoid natural products suggests new directions for discovery and/or engineering of the catalytic activity of those from labdane-related diterpenoid biosynthesis.

Terpenoids are the largest class of natural products, with more than 110,000 known (1), and are most frequently associated with plants, although they are more widely found. They can be first stratified by the number of isoprenyl units, ranging from isoprene to the polymers composed of greater than a 1000 such units found in natural rubber. Particularly well-known examples stem from the 10-carbon (2 isoprenyl unit) volatile monoterpenes that provide dominant scents such as limonene and pinenes, as well as the 30-carbon (6 isoprenyl unit) triterpenoid sterols that stabilize membranes and serve as key hormones (*e.g.*, the plant brassinosteroids). However, plants also use 20-carbon (4 isoprenyl unit) diterpenoid gibberellins as hormones. While sterols and hormones are required for normal growth and development, serving as primary metabolites, the vast majority of terpenoids serve more specialized roles and exhibit limited distribution. Nevertheless, at least in the plant kingdom, their biosynthesis can be traced back to these primary metabolites. This is perhaps most readily evident in the enzymes responsible for cyclization of the hydrocarbon backbones. For example, the terpene synthases involved in mono-, sesqui-, and di-terpenoid biosynthesis are generally derived from that required for gibberellin production, while the triterpenoid cyclases are derived from the cycloartenol synthase required for the production of phytosterols such as the brassinosteroids.

## Two classes of enzymes form terpenoid backbones

These two types of enzymes, termed class I terpene synthases and class II terpenoid cyclases, utilize distinct mechanisms to initiate their electrophilic reactions (2). Nevertheless, both catalyze what can be multi-step carbocation cascades in which cyclization and/or rearrangement can lead to over half of the carbons in the initial acyclic precursor changing bonding, hybridization, and/or configuration, despite the underlying reactivity limiting these reactions to the nanosecond timescale (3). Thus, unlike other natural products whose backbones are generally derived from iterative use of straightforward reactions, the generally stereo-dense hydrocarbon backbones underlying terpenoid variation are generated by reactions that are often extremely complex (4). The relevant enzymes have long been of interest based on their catalysis of some of the most complex known reactions (5). Given the multiple products often observed with these enzymes, there is particular interest in how they control their highly reactive carbocation cascades to mediate specific outcomes as would be optimal for their use as biocatalysts.

The class I terpene synthases catalyze lytic ionization of an allylic pyrophosphate ester bond, requiring a trio of divalent metal ions, prototypically Mg^2+^, that serve as co-substrates bound to the pyrophosphate (PP) moiety of the substrate (6). These 3 Mg^2+^ are bound by two characteristic motifs, both aspartate-rich, with the first conserved as **D**Dxx**D**, while the second is more variable but broadly conserved as (**N**/**D**)Dxx(**T**/**S**/G)xxx(**E**/**D**) (5). By contrast, the class II terpenoid cyclases initiate their reactions by protonation (2). While exemplified by the oxido-squalene cyclases, which protonate a terminal epoxide, other such enzymes initiate similar cyclization by protonation of a terminal olefin in their substrates, including the di-terpene cylases (DTCs) that characterize labdane-related diterpenoid (LRD) biosynthesis (7). Despite limited sequence similarity, class II terpenoid cyclases exhibit structural homology, being composed of a pair of double helical-barrel domains whose interface forms their active site (2). In addition, reflecting their analogous protonation of a terminal olefin, the DTCs share with squalene-hopene cyclases a characteristic Dx**D**D motif that cooperatively serves as the catalytic acid, with proton delivery from the ‘middle’ Asp, which is also conserved in oxido-squalene cyclases. Regardless of the initiation mechanism, these reactions are terminated by deprotonation, albeit this can be preceded by the addition of water, which generally yields hydroxylated products, although this further (potentially *via* olefin addition to the initially generated alkyloxonium) can lead to the formation of cyclic ether heterocycles (8).

## Unique biosynthesis of LRD backbones

**Figure 1:**
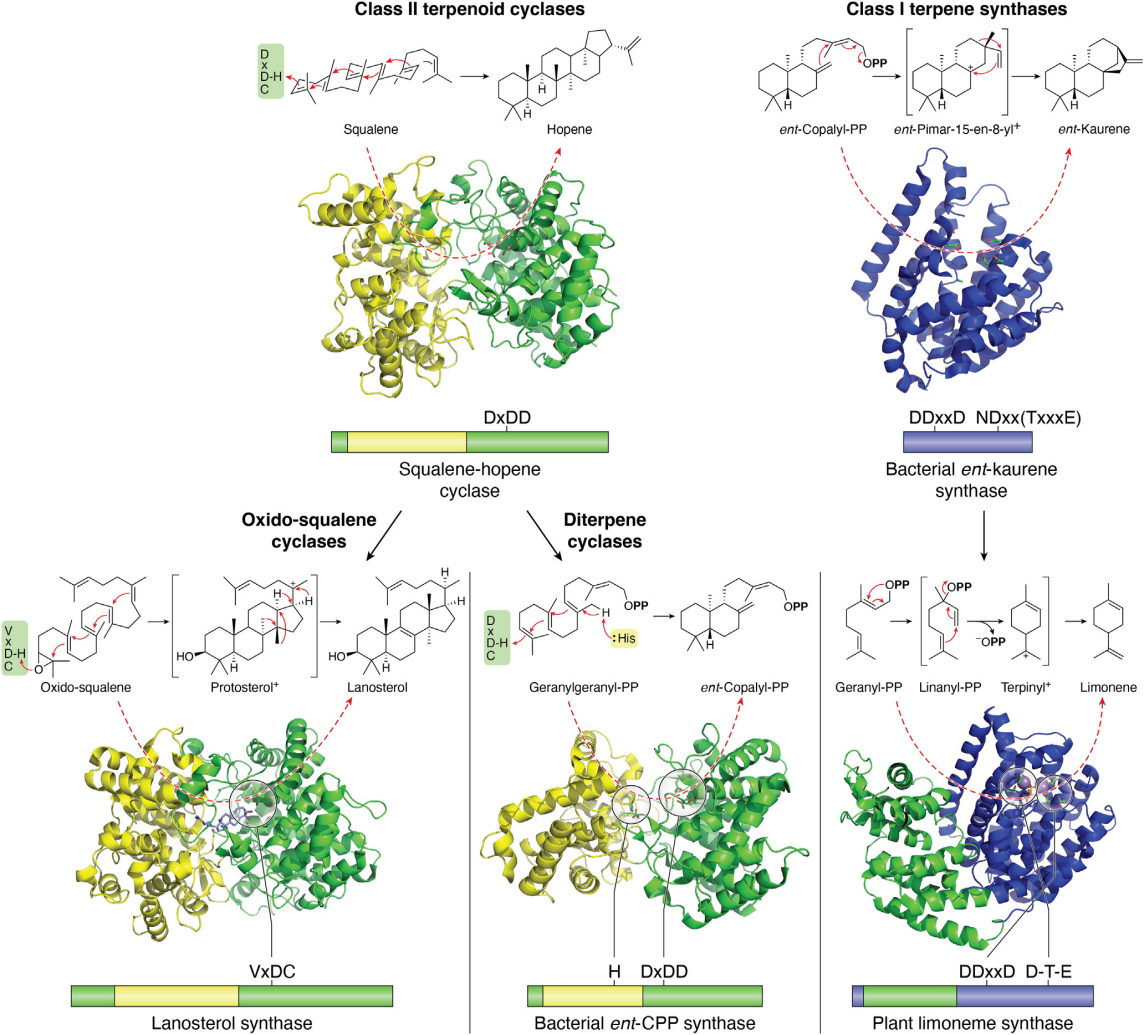
**Phylogenetic relationships and catalyzed reactions for class I terpene synthases and class II terpenoid cyclases.***Left*, class II terpenoid cyclases, composed of two helical domains termed β (*green*) and γ (*yellow*), which originated from (*top*) squalene-hopene cyclases, such as that depicted (97), which gave rise to oxido-squalene cyclases, such as the depicted (*bottom left*) lanosterol synthase with product (*purple*) (98), as well as diterpene cyclases such as the depicted (*bottom middle*) bacterial *ent*-CPP synthase (99). *Right*, class I terpene synthases, requiring only the catalytic α domain (*blue*), such as the depicted (*top*) bacterial *ent*-kaurene synthase (89), but can contain others such as the β domain (*green*), as depicted (*bottom*) for a citrus plant limonene synthase with substrate analog and trio of divalent metal ions (purple) (100), which is derived from diterpene synthases as described in the text. Also indicated are the key motifs/residues as described in the text.

While LRDs are defined by the use of DTCs in their biosynthesis (7), this is almost invariably followed by further transformation catalyzed by a (class I) di-terpene synthase (DTS). Indeed, DTCs specifically bicyclize the general diterpenoid precursor (*E,E,E*)-geranyl-geranyl-PP (GGPP), leaving intact the olefin adjacent to the pyrophosphate ester bond, thus preserving its allylic nature for subsequent lysis by a DTS. *Given the relationship of DTCs to other class II terpenoid cyclases and that of DTSs to other class I terpene synthases* (Fig. 1)*, study of their activity offers wider insight into terpenoid biosynthesis*. Indeed, these serve as model enzymes, not least as DTCs are soluble rather than membrane-associated, but also since they catalyze limited cyclization relative to that seen with triterpenoid cyclases (which form up to six rings). Moreover, the resulting bicycle decreases substrate flexibility (*versus* the usual acyclic terpene synthase substrates) for the subsequently acting DTSs. Although the DTC products have a reduced number of olefins relative to GGPP, which restricts the degree of cyclization, the variation in configuration (olefin/hydroxy positioning as well as stereochemistry) still offers substantial complexity for DTS catalysis. At the same time, comparison to the cyclization reactions catalyzed by related enzymes offers insight into the potential range of activity for DTCs and DTSs.

## The plant TPS family originated from a fused DTC-DTS

**Figure 2:**
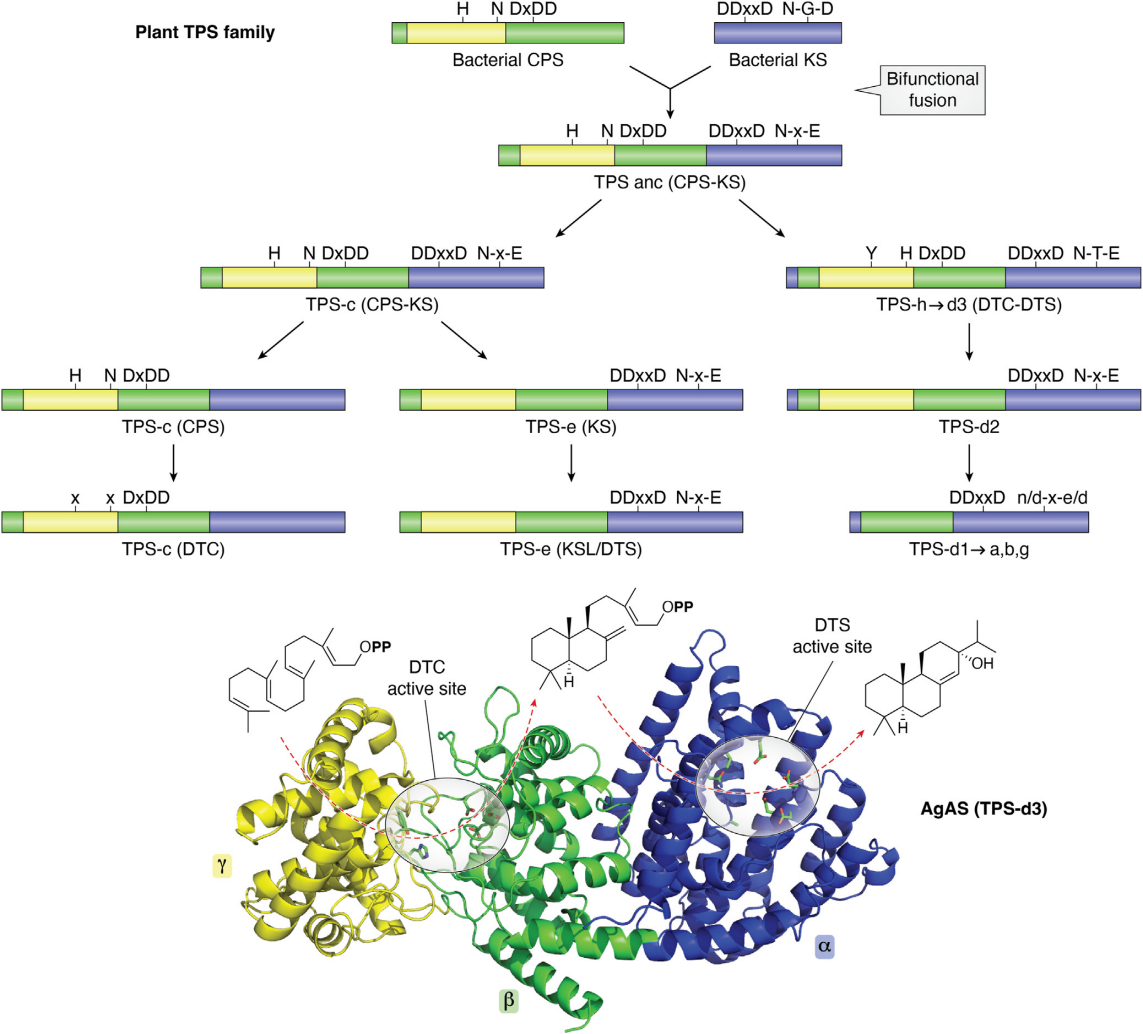
**Fused bifunctional DTC-DTS origin for the plant TPS family.***Top*, schematic representation of TPS evolution as recently described (14, 17), with relevant domains separately colored (α domain in *blue*, β domain in *green* and γ domain in *yellow*) and catalytic motifs/residues indicated as described in the text. *Bottom*, structure of the grand fir (*Abies grandis*) abietaenol synthase (42), AgAS from the TPS-d3 group, and its pair of sequentially catalyzed DTC and DTS reactions. Domains are colored as above. Side-chains for the residues defining each active site are shown. For the DTC active site this is the catalytic acid DxDD motif and catalytic base Y-H dyad. For the DTS active site this is the Mg^2+^-binding DDxxD and NDxxTxxxE motifs, along with a key alanine also described in the text.

It should be noted that the DTCs and DTSs are entirely unrelated, a point that has been somewhat confounded by the composition of the canonical plant terpene synthase (TPS) gene family (9) (Fig. 2). Specifically, because the TPSs seem to have originated from a bifunctional ancestor derived from the fusion of a (class II) DTC with a subsequently acting (class I) DTS, with such bifunctionality retained in some cases (Fig. 2). The interface between the relevant adjacent protein domains seems to have assumed sufficient structural importance (10), such that even long-standing sub-functionalization has retained these. For example, TPS sub-families that exhibit only class I activity in the relevant α-domain continue to contain at least the adjacent DTC domain (2), which has been designated β with the other then termed γ (11). While the primary sequence order is γβα, it should be noted that basic residues at the N-terminus seem to play a role in the C-terminal (α-domain) class I active site, both in the presence and absence of the γ-domain (10, 12), although in a few cases some activity can be seen with just the α-domain (13). In addition, even longer-standing (>350 million years) TPS lineages exhibiting only class II activity still contain the DTS α-domain (14). In either case, the catalytically irrelevant domains have not only lost the motifs associated with the missing activity but also are generally minimized to some extent (3). Thus, TPSs minimally contain both α- and β-domains, even when the β-domain is clearly catalytically irrelevant (*i.e.*, in mono-functional class I TPSs). Moreover, fungi also contain similar bifunctional (fused) DTC-DTSs, which have been hypothesized to originate from the plant TPSs (15), further conflating these two classes of enzymes. By contrast, these are prototypically separate in bacteria—*i.e.*, α-domain only terpene synthases and γβ-bidomain only class II terpenoid cyclases (note that oxido-squalene cyclases seem to be eukaryotic specific and are composed of only the γβ-bidomain in any case).

## The central role of gibberellin phytohormones in LRD evolution

LRDs are a large terpenoid superfamily, with over 7000 known (7), and are particularly prevalent in plants, where the production of *ent*-kaurene is required for gibberellin phytohormone biosynthesis (16), as this depends on a DTC to form *ent*-labda-8(17),13*E*-dien-15-yl (copalyl)-PP (*ent*-CPP), acting as a CPP synthase (CPS), with subsequent further cyclization by a DTS that then acts as a kaurene synthase (KS). Indeed, it has been hypothesized that the plant TPS family originated from a bifunctional CPS-KS, which was obtained from bacteria (17), with extant examples found in several non-seed plant species. The TPS family also contains bifunctional DTC-DTSs derived from early gene duplication and neo-functionalization (to more specialized LRD metabolism) of the ancestral CPS-KS, which gave rise to the TPS-a,b,d,g & h subfamilies (14). However, in most plant lineages, gene duplication and sub-functionalization has given rise to monofunctional CPSs and KSs, although these retain the ancestral γβα-tridomain architecture as well as specificity for production of *ent*-kaurene. In turn, these have undergone further gene duplication and neo-functionalization, leading to the production of more specialized LRD metabolites in many plant lineages (16). Altogether, these form the TPS-c and TPS-e/f subfamilies (respectively). While the repeated independent evolution of varied DTC and DTS activity from CPSs and/or KSs complicates interpretation of sequence conservation, several studies have identified residues whose identity is critical for such ancestral activity and, hence, provided insight into how these enzymes direct the catalyzed carbocation cascade to achieve specific product outcome (18, 19, 20, 21). Moreover, the divergent activities observed within the relevant subfamilies provide insight into the underlying enzymatic structure-function relationships, as described below.

## A model DTC and the importance of the catalytic base

**Figure 3:**
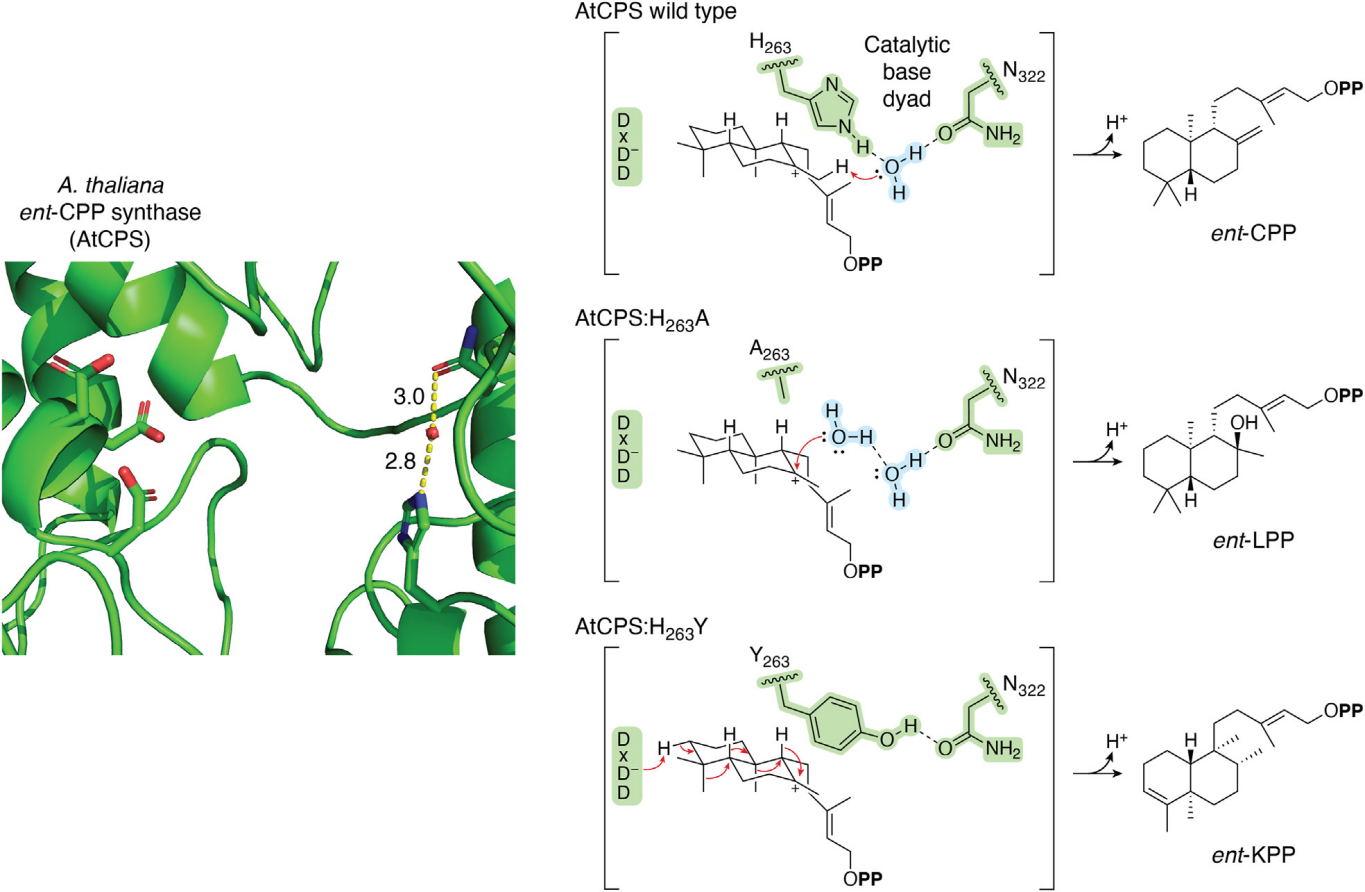
**Conserved catalytic base in phytohormone DTCs.***Left*, active site of *ent*-CPP synthase from *Arabidopsis thaliana* (AtCPS) with side-chains shown for designated residues from the DxDD catalytic acid motif, as well as histidine and asparagine, which help ligate a water, and compose the conserved catalytic base dyad in the CPSs involved in phytohormone (gibberellin) biosynthesis in either plants or bacteria. *Right, top*, hypothesized wild-type reaction mechanism, with well-coordinated water (two additional hydrogen-bonds to protein backbone not shown) serving as catalytic base to deprotonate *ent*-lambda-13-en-15-PP-8-yl carbocation intermediate from initial bicyclization. *Right, middle*, effect of alanine substitution for histidine (H263A), which leads to indicated addition of water and formation of *ent*-labda-13-en-8a-ol-15-PP (*ent*-LPP). *Right, bottom*, effect of tyrosine substitution for histidine (H263Y), which leads to indicated rearrangement and formation of *ent*-kolav-3,13-dien-15-PP (*ent*-KPP).

Reflecting both the status of *Arabidopsis thaliana* as the initial model plant for molecular studies, which led to early identification of its CPS, and the role of this AtCPS in gibberellin phytohormone biosynthesis, representing the ancestral class II TPS activity, AtCPS serves as a model DTC (22). Indeed, it was mutational analysis of the Dx**D**D motif in AtCPS, coupled to use of the more easily protonated oxido-GGPP substrate analog, that demonstrated these residues cooperatively serve as the catalytic acid initiating the carbocation-driven (bi)cyclization reaction catalyzed by DTCs (23). Moreover, determination of an x-ray crystal structure for AtCPS (24) led to further identification of the catalytic base that terminates the reaction leading to production of *ent*-CPP (Fig. 3). In particular, the structure revealed a histidine (H263) on the opposite side of the active site cavity from the DxDD motif, immediately suggesting this might serve as the catalytic base. Intriguingly, substitution with alanine (H263A) did not block activity but rather led to a novel product, *ent*-labda-13*E*-en-8α-ol-15-PP (*ent*-LPP), resulting from the addition of water to the initially formed bicyclic carbocation intermediate (*ent*-labda-13*E*-en-15-PP-8-yl^+^) before terminating deprotonation. Further inspection of the AtCPS structure revealed that H263 is hydrogen-bonded to water that is also hydrogen-bonded to an asparagine (N322), the substitution of Ala for which (N322A) also led to the production of *ent*-LPP (19). This water is further ligated by two additional hydrogen-bonds, one to the peptide backbone nitrogen of a lysine (K508) and the other to the peptide backbone carbonyl of N322 (25). In part as these interactions form a distorted rather than optimal tetrahedral arrangement, it has been hypothesized that this tightly constrained water serves as the catalytic base. Regardless, these Ala-substituted variants exhibit good catalytic efficiency and product specificity for *ent*-LPP.

It was latter realized that the catalytic base dyad found in AtCPS (H263 & N322) stem from a pair of small motifs conserved in all CPSs that act in phytohormone biosynthesis—*i.e.*, L**H**S and P**N**V—with Ala substitution for either leading to predominant production of *ent*-LPP in CPSs from a phylogenetically representative set of plants (21). Intriguingly, this holds true for the CPSs from plant-associated bacteria that produce gibberellins (26, 27, 28, 29, 30, 31, 32), consistent with a bacterial origin for the TPS gene family (21). However, these motifs are not necessary for production of *ent*-CPP, as other such DTCs utilize alternative arrangements (also verified by mutational analysis), supporting their more specific association with the ancestral (phytohormone) activity (21). Indeed, the broad conservation of these motifs appears to be predictive, further supporting such CPS activity in the TPS ancestor (14).

## DTC product outcome varies with catalytic base

By contrast, in at least some cases variation in these motifs can predict alternative DTCs product outcome. Perhaps the most striking example of this stems from additional mutational analysis of AtCPS, wherein substitution of phenylalanine or, especially, tyrosine for H263 led to production of the clerodane precursor *ent*-kolav-3,13*E*-dien-15-PP (*ent*-KPP), resulting from a series of four 1,2-shifts, alternating between hydride and methyl substituents, around the decalin bicycle, with removal of the proton originally added to initiate cyclization (Fig. 3), presumably by the same Asp (33). Again, these variants exhibit good catalytic efficiency and product specificity. Indeed, the latter discovery of several native *ent*-KPP synthases found these contained either Phe or Tyr at this position, and in two independently evolved cases substitution of His for these was found to lead to the production of *ent*-CPP (34, 35). Particularly, given such shuffling of the decalin substituents necessitates cation intermediates located at additional carbons beyond those required for cyclization, these results highlight both conservation of the inert nature of this region of the DTC active site (*i.e.*, that within which the carbocation cascade occurs) as well as the importance of catalytic base positioning (or absence) for termination of the reaction and dictating product outcome.

In other cases, DTCs with alternative product outcomes these motifs vary but are otherwise conserved. For example, from the bifunctional (DTC-DTS) TPSs involved in conifer resin acid biosynthesis (*i.e.*, the TPS-d3 group), where early identification led to the abietaenol synthase from grand fir (*Abies grandis*, AgAS) serving as another model system for both DTC and DTS activity (10, 36, 37, 38, 39, 40, 41). The DTC activity of these enzymes leads to (normal) CPP, and the determination of a crystal structure for AgAS [(42), see Fig. 2] led to identification of the relevant catalytic base. Intriguingly, these correspond to modification of the ancestral (CPS) motifs, now conserved as L**Y**S and PC**H**, with direct hydrogen bonding between the Tyr and His side chains observed (43). The role of this distinct dyad as the catalytic base has been indicated, in part, as substitution of Ala or, particularly, Asp [as guided by identification of an LPP-producing TPS-d3 group member (44)] for the His (45), or Phe for the Tyr (43), leads to incorporation of water and, hence, production of LPP. Moreover, other variations in these motifs leading to alternative arrangements of residues that seem to serve as the catalytic base are associated with changes in product outcome in at least DTCs from the Lamiaceae plant family (46). Nevertheless, in certain cases, residue(s) at other positions serve as the catalytic base (47). Again, this emphasizes the importance of catalytic base positioning for termination of the reaction in determining product outcome.

## A broad range of residues can serve as catalytic bases

Given the emphasis placed on catalytic base positioning by the results described above, it should be noted that unactivated hydroxy groups or carbonyls can deprotonate even relatively stable tertiary carbocations—*i.e.*, based on their relative pK_a_ of greater than −4 or −7 *versus* less than −10 (respectively). This is highlighted by a recent analysis of the DTC producing halima-5,13*E*-dien-15-PP (HPP), reflecting partial decalin substituent shuffling (with the initial methyl migration characterizing the halimane backbone), from *Mycobacterium tuberculosis* (MtHPS), wherein structural resolution *via* x-ray crystallography was combined with a computational approach to suggest Y479 acts as the catalytic base (48). Subsequent mutational analysis supported this assignment, with substitution of Phe leading to production of labda-8,13*E*-dien-15-PP, which further computational work indicates results from deprotonation by a peptide backbone carbonyl (49). While recognizing this requires the relevant functional group to exhibit optimal geometry (orientation and distance), these results further stress the importance of shielding in enzymatic active sites for carbocation cascade reactions.

## DTC reactant positioning matters

The importance of catalytic base positioning includes reactant orientation, which can be altered with the use of distinct divalent metal ions, as was recently emphasized by observation of a subtle but significant change in DTC activity of the bifunctional premutilin synthase from the fungus *Clitopilus passeckerianus* (CpPS) (50). Note that these divalent metal ions (almost invariably Mg^2+^) seem to be co-substrates brought in by interaction with the pyrophosphate moiety, exhibiting only minimal interaction with the DTC active site. While this was not evident in the AtCPS structure, comparison with other class II cyclase structures indicates DTCs only interact with these co-factors/substrates *via* a conserved glutamate (51). Particularly as there has been some confusion about its location (11, 52), it is worth noting that in the plant TPS family, this Glu is found in a conserved GF**E** motif (*e.g.*, E211 in AtCPS), while in bacterial DTCs the associated motif is (G/A)x**E** (*e.g.*, E120 in MtHPS).

## DTCs can vary ring structure

**Figure 4:**
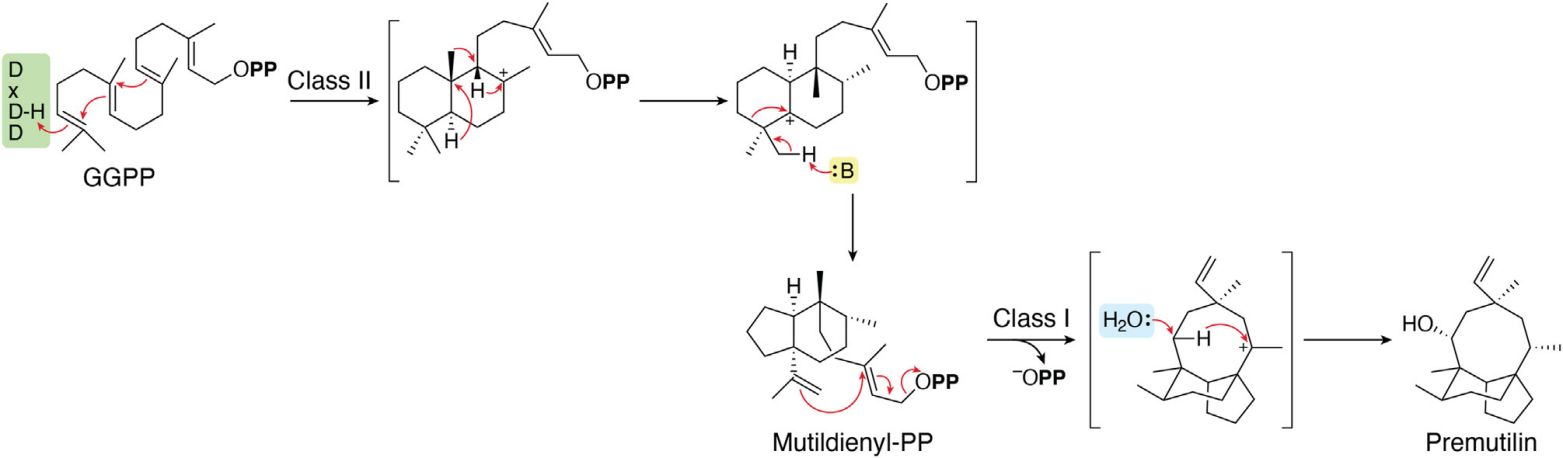
**Unusual DTC and DTS reactions from pleuromutilin biosynthesis.** These reactions represent unique transformations, with the relevant DTC (class II) cyclization (*top*) requiring ring contraction, while the relevant DTS (class I) cyclizes a non-labdane substrate (*bottom*), yielding a macrocycle, with addition of water prior to deprotonation leading to a hydroxylated final product.

Intriguingly, the DTC active site of CpPS produces *syn*-mutil-4(19),13*E*-dien-15-PP (*syn*-MPP), whose 5-6 bicycle results from ring contraction of a *syn*-halima-13*E*-en-15-PP-5-yl^+^ intermediate (Fig. 4), rather than the more common final methyl migration leading to a clerodane type product (53). Indeed, quantum chemical calculations indicate ring contraction is energetically more favorable, which suggests that simply placing a base capable of deprotonating the relevant methyl might be sufficient to enable such product outcome (54). Regardless, this highlights the ability of DTCs to generate non-decalin bicycles, significantly increasing the range of potential products. In addition, the ability of the DTS active site in CpPS to carry out further cyclization of the highly rearranged *syn*-MPP (relative to the otherwise exclusive cyclization of only labdane-type DTC products) also highlights the structural complexity that can be generated by the sequential cyclase and synthase reactions that initiate LRD biosynthesis (55, 56).

## DTSs and the Mg^2+^ binding motifs

DTSs contain the 2 Mg^2+^-binding motifs characteristic of all (class I) terpene synthases (6). Indeed, the appearance of glycine in the ‘middle’ position of the (**N**/**D**)Dxx(**T**/**S**/G)xxx(**E**/**D**) motif was first noted in plant KSs and explored with AgAS (57). Moreover, there is further variation at this position as, just from plant DTSs, glutamine, and Ala also have been observed [*e.g.* (58)]. However, arguably more intriguing is the variation at the ‘first’ position, as this alters charge and, hence, electrostatic interactions with the pyrophosphate anion co-product, which has been hypothesized to serve as a general base (and sometimes acid) in terpene synthase catalyzed reactions (59). Although this residue is always Asn in DTSs (including KSs) from both plants and bacteria, it varies in terpene synthases more widely, and its identity has been correlated with the ability of the pyrophosphate anion co-product to act as a catalytic acid at later steps in the carbocation cascade reaction (60).

## Rice as a model system for investigating DTS activity

Rice (*Oryza sativa*) was known to use multiple LRDs as phytoalexins and, building on the early sequencing of its genome reported in 2002 (61, 62), has become a model system for investigating such biosynthesis (63). As predicted (64, 65), the first *syn*-CPP synthase was identified from this cereal crop plant (66, 67). More broadly, the utility of focusing on a single species was validated by early results with the relevant DTSs. These all are closely related to KSs and, hence, termed KS-like (KSL), falling within the TPS-e subfamily that then contains both KSs and such KSLs (68). Notably, investigation of the OsKSLs dramatically expanded identified plant DTS activity beyond the KSs and AgAS, which were the only known at that time.

## Short-circuiting a complex carbocation cascade

Strikingly, study of the rice KSLs further led to the discovery of a single residue switch for product outcome that is widely applicable to all plant KSs. This finding serendipitously arose from competing studies that worked with distinct subspecies (ssp.), leading to two different activities assigned to OsKSL5, with the allele from ssp. japonica (OsKSL5j) specifically producing *ent*-pimara-8(14),15-diene (69), while that from ssp. indica (OsKSL5i) specifically produced *ent*-isokaurene (a double bond isomer of *ent*-kaurene), despite their 98% amino acid (aa) sequence identity, matching the activity exhibited by the closely related OsKSL6 (70). This difference in product outcome was then tracked down to the identity of a single residue, with isoleucine associated with the production of *ent*-isokaurene while substitution of Thr (as found in OsKSL5j) in either OsKSL5i or OsKSL6 results in the production of *ent*-pimaradiene instead (18). Strikingly, the converse use of this switch to increase reaction complexity applies not only to OsKSL5j, but also to the *syn*-pimara-7,15-diene synthase OsKSL4, as substitution of Ile for Thr at this position leads to predominant production of the further cyclized and rearranged *syn*-aphidicolene with only small amounts of the original tricycle formed (71).

**Figure 5:**
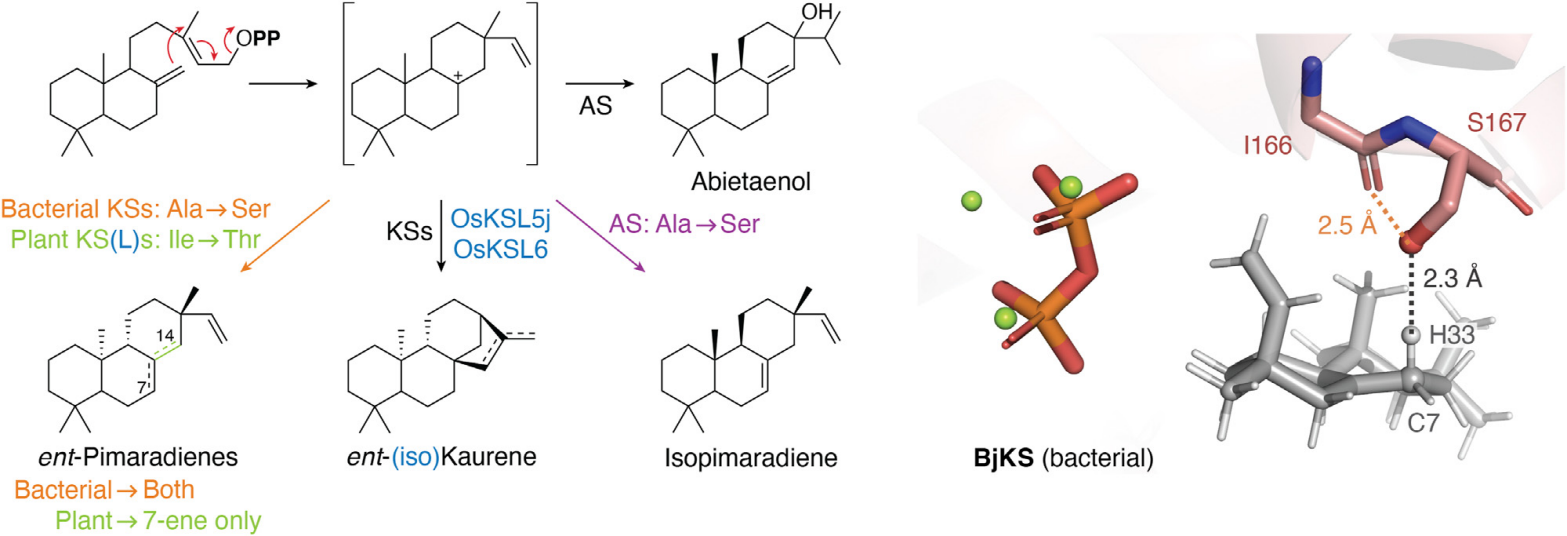
**Introduction of a hydroxyl containing residue can short-circuit carbocation cascades.***Left*, as found for Thr substitution for key Ile in plant KS(L)s and Ser substitution for key Ala in bacterial KSs (from gibberellin biosynthesis), as well as in abietaenol synthases (AS) from the TPS-d3 group. *Right*, results of computational (TerDockin) analysis of the *ent*-kaurene synthase from the bacterial gibberellin producing *Bradyrhizobium japonicum* (BjKS) indicating introduced hydroxyl group acts a catalytic base [depicted here is only that for deprotonation at carbon-7 (C7) and, hence, production of *ent*-pimara-7,15-diene, although A167S mutant produces both roughly equal amounts of this and *ent*-pimara-8(14),15-diene, with TerDockin indicating the hydroxyl also deprotonates C14 just as well] (90).

The production of pimaradienes in these studies results from deprotonation of the pimar-15-en-8-yl^+^ formed by initial cyclization (Fig. 5), and it is now hypothesized that the Thr hydroxyl serves as the relevant catalytic base. In its absence, the inert nature of the aliphatic Ile side-chain allows a more complex reaction, potentially driven (in part) by carbocation migration towards the pyrophosphate anion co-product that presumably serves as the catalytic base for the production of *ent*-(iso)kaurene (18), as has been hypothesized in many (class I) terpene synthases (59). This highlights the importance of catalytic base positioning (or introduction) as well as the inert nature of the class I terpene synthase active site, much as described above for class II terpenoid cyclases.

Strikingly, the presence of Ile at this position was conserved in the rice KS (OsKS1) as well as that from *A. thaliana* (AtKS), with substitution of Thr leading to a dramatic analogous change in product outcome—*i.e.*, to almost exclusively *ent*-pimara-8(14),15-diene (18). Indeed, it was later realized that this Ile is conserved in a P**I**x motif in all plant KSs, with substitution of Thr resulting in a similarly efficient switch to production of *ent*-pimara-8(14),15-diene (20). Just as with the CPS catalytic base dyad motifs also associated with phytohormone biosynthesis, the presence of the P**I**x motif has proven to be predictive, consistent with such KS activity in the ancestral TPS—*i.e.*, this was a bifunctional CPS-KS (14).

## Extending the introduction of a hydroxyl to short-circuit complex reactions

The original work with OsKSL5 also immediately led to the discovery of a similar switch for product outcome in the class I active site of the bifunctional DTC-DTSs from the TPS-d3 group. This was based on identification of isopimara-7,15-diene and abieta-8(14)-en-13-ol [originally thought to be a mixture of abietadienes (72)] synthases from *Picea abies* (PaPS and PaAS, respectively) that shared 91% aa sequence identity (73). Mutational analysis of AgAS was then undertaken, focused on a specific residue, which sequence alignment suggested was 4 residues (*i.e.*, one helical turn) upstream of the key Ile in the plant KSs and is similarly conserved—*i.e.*, as Ala in abietane-forming synthases (*e.g.*, AgAS & PaAS) but was Ser in PaPS. Indeed, substitution of Ser for this Ala in AgAS resulted in primary production of isopimara-7,15-diene, similarly representing deprotonation of the isopimar-15-en-8-yl^+^ formed by initial cyclization prior to the rearrangement leading to the abietane backbone (74). However, with PaPS substitution of Ala for the corresponding Ser only marginally affected product outcome and it was found that exchange of 1 to 3 more active site residues was required for increasingly specific production of abietaenol (75). Nevertheless, substitution of Ser for the key Ala in PaAS was similarly effective in short-circuiting the usually catalyzed more extensive carbocation cascade reaction (*i.e.*, as seen with AgAS), although exchanging the additional two or three residues demonstrates epistatic effects between these and the key Ala/Ser position, with incremental but noticeably increased fidelity of isopimara-7,15-diene production (75).

## Epistatic effects on key residues

Further work with OsKSL5 led to the discovery of a residue with a similar epistatic effect on product outcome. Specifically, as substituting Ile for the key Thr in the *ent*-pimaradiene synthase ortholog OsKSL5j was not sufficient to convert this to specific production of *ent*-isokaurene, with significant amounts of *ent*-atiserene as well as small amounts of *ent*-kaurene also produced (18). However, additional exchange of a nearby residue, from valine to leucine, enabled specific production of *ent*-isokaurene (76). The converse substitution of Val for the corresponding Leu in the *ent*-isokaurene synthase ortholog OsKSL5i similarly led to less specificity, with analogous production of *ent*-atiserene and *ent*-kaurene observed, but the addition of which did not affect the production of *ent*-pimaradiene by the Ile to Thr switch. Intriguingly, the presence of Leu in this position was further found to be conserved in plant KSs more generally. Although substitution of Val for this Leu did not affect KS product outcome on its own, when combined with the Ile to Thr switch this led to decreased specificity, including production of significant amounts of 8α-hydroxy-*ent*-pimara-13-ene, derived from addition of water to the *ent*-pimar-15-en-8-yl^+^ intermediate prior to deprotonation (76).

In the case of the TPS-d3 group, the AgAS crystal structure (42) revealed the key Ala/Ser product ‘switch’ residue sits at the G1/G2 helix-break, with the side-chain protruding into the DTS active site (see Fig. 2). This supports the hypothesis that the Ser hydroxyl potentially acts as a base to short-circuit the reaction, directly yielding pimaradiene, while the epistatic residues presumably act more indirectly as they generally fall outside the active site. Unfortunately, there are no experimentally determined structures for any member of the KS(L)/TPS-e subfamily, which limits interpretation of the mutational results described above—*e.g.*, does the introduced hydroxyl group from Thr substitution for the key Ile act as a catalytic base? However, an obvious inference from the KS(L) work is that the epistatic Val for Leu substitution increases the size of the active site cavity, thereby reducing specificity as well as enabling the presence of water in an appropriate position for addition to *ent*-pimar-15-en-8-yl^+^. Consistent with this latter part of the hypothesis, at least with AtKS it was found that further opening up the active site by substituting Ser instead of Thr for Ile alongside the Val for Leu replacement leads to a more specific production of 8α-hydroxy-*ent*-pimara-13-ene (76).

## Room for further insights from rice KSLs

The variation in OsKSL activity between distinct subspecies of rice has since been extended beyond OsKSL5. For example, there is surprising divergence at the genetic loci for *OsKSL8/9*, with at least four functionally distinct variants observed (77). Unfortunately, the divergence between these has stymied the identification of the key changes leading to their distinct product outcome. On the other hand, it was recently reported that most alleles of OsKSL10 produce *ent*-miltiradiene rather than the *ent*-sandaracopimaradiene produced by that first characterized from the originally sequenced ssp. japonica cultivar Nipponbare (78), with this alternative product outcome now assigned to a single residue switch from Ala to Gly (79). Thus, particularly with the large numbers of sequenced genomes for a variety of cultivars (80), as well as closely related wild-rice species (81), along with the increasingly numerous studies of phylogenetically related but often mechanistically distinct KSLs from other grasses/cereals (82), the continued utility of focusing on rice as a model system for such evolution-guided investigation of enzymatic structure-function relationships is evident.

## The known range of DTS catalysis

**Figure 6:**
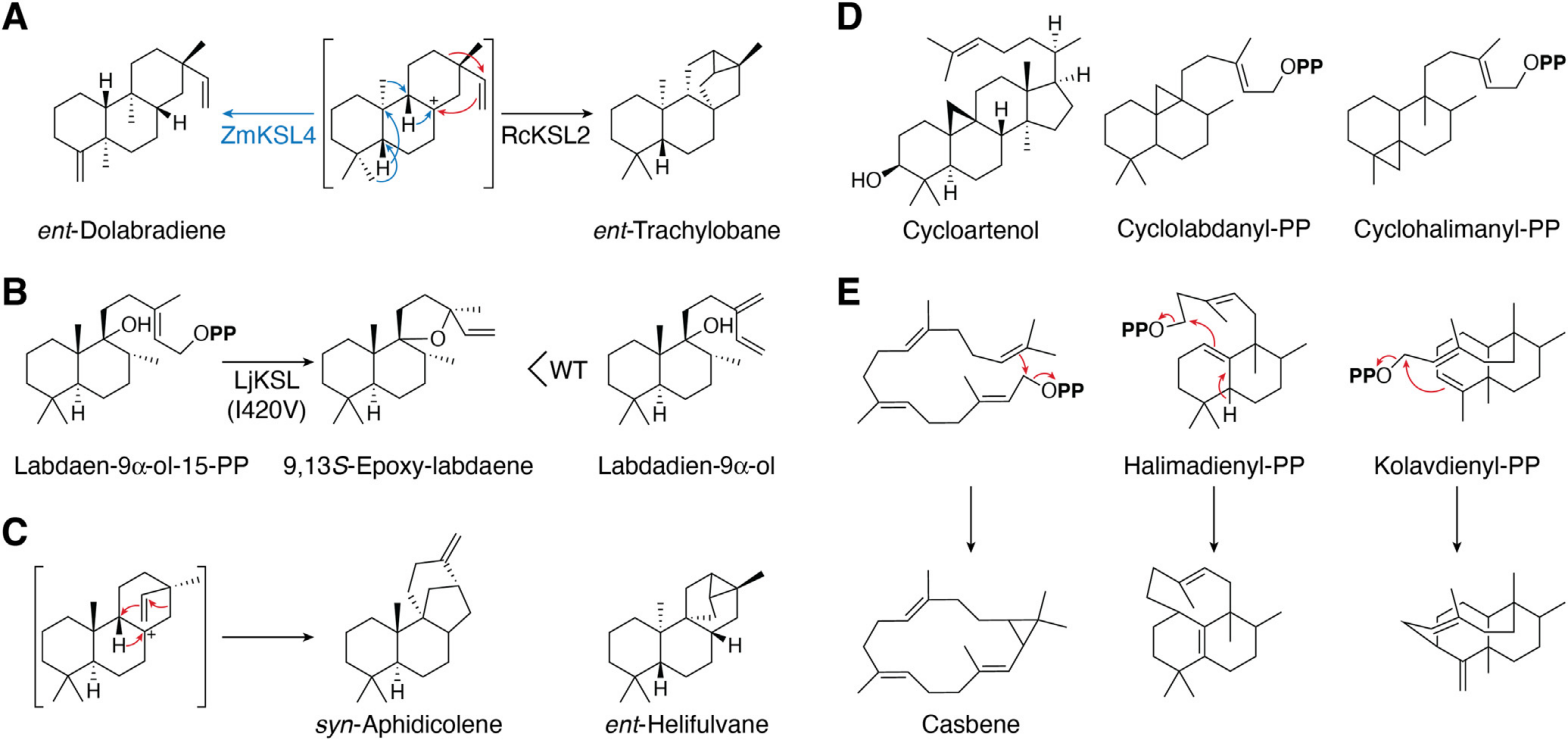
**Known and homolog comparison predicted reaction capacities.***A*, range of known DTS reactions exemplified by *ent*-trachylobane (maximal cyclization) as produced by the castor bean RcKSL4, and dolabradiene (full decalin substituent shuffling) as produced by the maize ZmKSL4, shown here. *B*, specificity of heterocyclization can be increased as shown for a KSL from *Leonurus japonicus* (LjKSL), where the wild-type (WT) makes primarily labdane-9α-ol, along with an epimeric mixture of 9,13-epoxy-labdaenes, while the I420V mutant makes exclusively 9,13*S*-epoxy-labdaene (88). *C*, substrate stereochemistry impacts secondary cyclization pattern, with 9,16-ring closure exclusively used with *syn*-CPP, such as shown for formation of *syn*-aphidicolene (*left*), while 8,16-cyclization, as seen in formation of *ent*-kaurene (or trachylobane), is utilized with normal/*ent*-CPP, although it has been suggested that *ent*-helifulvane (shown), which requires 9,16-cyclization, might be produced by a DTS (95). *D*, production of cycloartenol by oxido-squalene cyclases suggests DTCs should be capable of analogous cyclopropanyl ring production such as those shown. *E*, cyclization to primary position of allylic carbocation by other terpene synthases, such as shown here for production of casbene (*left*), suggests DTSs should be capable of such (macro)cyclization, which would enable cyclization of a broader range of DTC products, such as those shown here.

Beyond the macrocyclization catalyzed by CpPS with mutildienyl-PP, all known DTS cyclization reactions seem to otherwise be restricted to labdane (rather than halimane or clerodane) type substrates, almost entirely with CPP (of any stereochemistry). Nevertheless, there are a range of potential DTS products from reaction with CPP (Fig. 6*A*), which is exemplified at one extreme by trachylobane, which contains the maximum number of rings – *i.e.*, one more than the number of olefins in the substrate, with the ‘extra’ from deprotonation of the transition state for the alkyl migration (ring rearrangement) otherwise leading to *ent*-kaurene. RcKSL2 from castor bean (*Ricinus communis*), which forms this pentacycle from *ent*-CPP, provides an example of such a DTS – *i.e.*, one catalyzing tri-cyclization (58). The other extreme is exemplified by dolabradiene, resulting from initial cyclization to a pimar-13-en-8-yl^+^ intermediate followed by analogous shuffling of the substituents all the way around the decalin bicycle as seen with clerodane-producing DTCs. ZmKSL4 from maize (*Zea mays*), which catalyzes such a reaction with *ent*-CPP, provides an example of this activity—*i.e.*, cyclization and full substituent rearrangement (83).

DTSs also readily catalyze heterocyclization with hydroxylated DTC products—*e.g.*, even KSs efficiently react with *ent*-LPP (which resembles their native substrate) to produce *ent*-manoyl oxide (43). Similarly, from the alternatively hydroxylated labda-13*E*-en-9α-ol-15-PP, it has been found that DTSs can catalyze spiro-epoxy ring formation, as required for the biosynthesis of derived LRDs in Lamiaceae plant species. However, these are surprisingly non-specific, producing a mixture of not only the two C13 epimers but also non-cyclized derivatives (84, 85, 86). Notably, in at least one case mutational analysis of the previously identified DTS ‘switch’ region (around the G1/G2 helix break), particularly of a residue already known to affect product outcome in such Lamiaceae KSLs (87), led to identification of a single residue substitution leading to specific production of 9,13*S*-epoxy-labda-14-ene with good catalytic efficiency (Fig. 6*B*) (88).

## Going outside plants and applying the TerDockin computational approach

While most DTCs and DTSs are found in plants, examples can be found in microbes (*e.g.*, the fungal CpPS and bacterial MtHPS). Perhaps most striking are those involved in the production of gibberellins by plant-associated bacteria, which form a large, conserved family (26, 27, 28, 29, 30, 31, 32), offering some of the same advantages for evolution-guided study of enzymatic structure-function relationships as noted above for certain activities/groups within the TPS family. For example, as noted earlier, there is a conservation of the catalytic base dyad between plant CPSs involved in gibberellin biosynthesis and these functionally analogous bacterial CPSs (21). Although such conservation is not found between the plant and bacterial KSs, a similar single residue switch was identified. Specifically, a highly conserved Ala that the available crystal structure for the KS from *Bradyrhizobium japonicum* (BjKS) indicates is analogous to the DTS product ‘switch’ residue in AgAS—*i.e.*, sits at the G1/G2 helix-break (89). The alignment further suggests this also is 4 residues upstream of the key Ile in the plant KSs. Indeed, the substitution of Ser for this Ala similarly led to short-circuiting of the reaction, albeit with a roughly equal mix of *ent*-pimara-7,15-diene and *ent*-pimara-8(14),15-diene observed (90). Notably, application of the TerDockin computational approach, combining quantum chemical analysis [validated for terpene synthase reaction mechanisms (91)] with (bio)chemically-constrained docking of the resulting carbocations *via* the Rosetta Molecular Modeling Suite (92), indicates that the introduced Ser deprotonates the initially formed *ent*-pimara-13-en-8-yl^+^ at two alternative positions consistent with the observed ratios of the pair of products (Fig. 5). Thus, at least in this BjKS:A167S mutant, it seems that the introduced hydroxyl group, potentially with activation from the free peptide backbone carbonyl of the preceding residue (*i.e.*, given the helical break; see Fig. 5), acts catalytically as a base (90).

The TerDockin approach has also recently been shown to apply to DTCs, specifically for the identification of a Tyr acting as a catalytic base in MtHPS, as described above (49). It will be of interest to further apply TerDockin to the structurally resolved AtCPS (*e.g.*, does the ligated water serve as the catalytic base and how do Ala substitutions lead to the incorporation of water and production of *ent*-LPP operate?). Similarly, the known structure of AgAS enables analogous analysis of not only the Tyr-His catalytic base dyad in the DTC active site (*e.g.*, which residue serves as the catalytic base and how do the known substitutions leading to the incorporation of water and production of LPP operate?), but also the Ala/Ser product “switch” in the DTS active site (*i.e.*, does the introduced hydroxyl act as a catalytic base?).

Although no structures have yet been determined for any member of the KS(L)/TPS-e subfamily, simple alignment suggests the Ile/Thr product ‘switch’ in plant KSs should simply sit one helical turn deeper in the active site. However, the composition of the associated P**I**x motif offers an intriguing alternative. Specifically, the proline would be expected to act as a helix-breaker, which could offer similar activation of the hydroxyl group from the substituted Thr as TerDockin indicates for introduced Ser in BjKS. Determination of a plant KS(L) structure will elucidate this potential shift or extension of the G1/G2 helix-break. Regardless, now that TerDockin has been validated by retrospective analyses of previously reported mutational results and found to enable the identification of key catalytic residues, it can be anticipated that this approach will be adapted to drive prospective application (*i.e.*, rational engineering of product outcome), not least increased specificity as well as potentially also catalytic efficiency.

## Testing the limits of DTS/DTC catalysis

Rational engineering offers the opportunity to probe the puzzling observation that the nature of DTS catalyzed bicyclization seems to depend on stereochemistry of the original CPP substrate. Specifically, further (secondary) cyclization of the (iso)pimaraenyl carbocation intermediate formed by initial cyclization, which seems to only occur following a 1,2-hydride transfer with *syn*-reactants, leading to 9,16-ring closure (*e.g.*, Fig. 6*C*), while with normal/*ent*-reactants this occurs directly—*i.e.*, *via* 8,16-ring formation. Quantum chemical analysis indicates the alternative secondary cyclization pattern is accessible for each stereoisomer (93, 94). Accordingly, such ‘non-native’ product outcome should be possible. Indeed, 9,16-cyclization of *ent*-isopimaraenyl^+^ must occur in the production of *ent*-helifulvane (95).

Even more fundamentally, such engineering provides an opportunity to investigate the interoperability of DTC and DTS conservation with other class II terpenoid cyclases and class I terpene synthases, respectively. For example, all plants have an oxido-squalene cyclase that produces cycloartenol, which contains a cyclopropyl resulting from deprotonation of the transition state for a 1,2-methyl migration (Fig. 6*D*), and it has been possible to engineer SHC to produce a monocycle (96). Thus, although no DTCs catalyzing such reactions are currently known, they may well exist, and it should be possible to engineer such activity in any case. Similarly, the restriction of DTS catalyzed cyclization to reactions with labdane-type DTC products, almost entirely CPP, reflects the observation that these occur by addition to the tertiary position of the allylic carbocation formed by initiating ionization, which limits the “reach” of the isoprenyl-PP tail. However, the precedent set by other terpene synthases indicates that it should be possible to add to the primary position, either directly or following isomerization of the pyrophosphate to the tertiary position (3), to enable cyclization with other (halimane or clerodane) type substrates as well. Again, while no DTSs catalyzing such reactions are currently known, it can be speculated that relevant DTSs might naturally exist or could be engineered (*e.g.*, Fig. 6*E*). Although this would require extensive changes in the active site to accommodate the necessary shift of the decalin bicycle relative to the conserved Mg^2+^ and, hence, substrate pyrophosphate binding site, the macrocyclization catalyzed by the DTS active site in the fungal CpPS demonstrates such re-positioning is possible (c.f., Figs. 4 and 6*E*).

## Summary

In conclusion, study of DTCs and DTSs has provided insights applicable to terpenoid biosynthesis more broadly, specifically the carbocation cascade reactions responsible for formation of the hydrocarbon backbones that underly their intricate structural diversity. This includes elucidation of the origins and subsequent evolution of the TPS family in plants, where the bulk of terpenoid natural products are found. Notably, this origin from a CPS-KS implies that the promiscuity for which this enzymatic family is particularly notorious must have devolved from the specificity associated with such ancestral phytohormone biosynthesis. While such specificity has long been recognized to depend on restricting the potential configurations of the substrate and hence reactant, the investigations of DTCs and DTSs reviewed here highlight the importance of catalytic base positioning. Moreover, this review further emphasizes the advantages of investigating model systems that enable evolution-guided analysis of the relevant enzymatic structure-function relationships. It should be noted that insights into these complex reactions are perhaps most readily derived from the study of enzymes that exhibit strict specificity, as described here, simplifying interpretation of the results from mutational analysis. Regardless, study of DTCs and DTSs has already provided impactful insights into enzymatic control of carbocation cascade reactions, including engineering of increased specificity, and can be expected to continue doing so in the future.

## Data availability

All data described are contained within the article.

## Conflict of interest

The author declares that he has no conflicts of interest with the contents of this article.
